# Literary Notes

**Published:** 1898-10

**Authors:** 


### LITERARY NOTES.

---

Mr. W. B. Saunders, medical publisher, Philadelphia, has in process of publication a series of hand atlases that are of a remarkable character. They embrace almost every department of medicine and are well calculated to aid in the elucidation of many intricate subjects. They are offered at a remarkably low price. This is partially if not wholly explained by the fact that the initial cost of publication in the making of the expensive colored plates is borne by eleven publishers instead of one, as is usually the case, thus making it possible to produce them at a price below their apparent value.

---

The *Southern Medical Exchange* is the name of a new periodical that appeared in September. It is published at Atlanta, Ga., and is devoted especially to the trade interests of the physician and druggist and is said to be the only enterprise of its kind in the Southern States.

---

THE Bulletin of the Harvard Medical Alumni Association, Number 12, contains the report of the eighth annual meeting, held at Boston, June 28, 1898. Besides the constitution and proceedings, in it is published a full report of the annual dinner, including speeches. This is by far the handsomest and most complete report of a medical alumni association that comes to our table, and its annual appearance in beautiful tint and type is a delight to the reader and a credit to its sponsors.

